# Instability by Extension of an Elastic Nanorod

**DOI:** 10.3390/nano15221689

**Published:** 2025-11-07

**Authors:** Armin Berecki, Valentin Glavardanov, Sanja Mihok, Nenad Grahovac, Miodrag Zigic

**Affiliations:** Department of Mechanics, Faculty of Technical Sciences, University of Novi Sad, Trg D. Obradovica 6, 21000 Novi Sad, Serbia; armin@uns.ac.rs (A.B.); vanja@uns.ac.rs (V.G.); sanjao@uns.ac.rs (S.M.); mzigic@uns.ac.rs (M.Z.)

**Keywords:** nanorod, extension, two-phase local/nonlocal stress-driven model, buckling, post-buckling, Liapunov–Schmidt method

## Abstract

The static stability of an elastic, incompressible nanorod subjected to an extensional force is analyzed. The force is applied to a rigid rod that is welded to the free end of the nanorod. The material behavior of the nanorod is described using a two-phase local/nonlocal stress-driven model. Mathematically, the problem is formulated as a system of nonlinear differential equations suitable for nonlinear analysis. For the analysis, the Liapunov–Schmidt method is employed. Depending on a geometric parameter (the length of the rigid rod) and nonlocal parameters (the small length-scale parameter and the phase parameter), the buckling load and post-buckling behavior of the nanorod are determined. The results show that the nonlocal effect increases the buckling load, indicating a stiffening effect. An increase in the length of the rigid rod decreases the buckling load. Regarding the post-buckling behavior, it is shown that both supercritical and subcritical bifurcations can occur, depending on the values of the geometric and nonlocal parameters. The occurrence of a subcritical bifurcation, which is highly undesirable in real-world constructions, is a novel effect not observed in the classical Bernoulli–Euler theory.

## 1. Introduction

Nanomaterials represent an exceptional class of materials whose emergence and applications have profoundly transformed numerous fields of science and engineering. In general, nanomaterials are defined as materials possessing at least one dimension in the range of 1–100 nm. The conceptual foundation of nanoscience was established by Richard Feynman in his seminal lecture “There’s Plenty of Room at the Bottom” where he proposed that the ability to manipulate atoms and molecules individually could fundamentally reshape the scientific landscape [1]. Since the discovery of multiwalled carbon nanotubes in 1991, research in nanoscience has expanded rapidly, encompassing both synthesis techniques and practical implementations. Today, nanomaterials are a central topic in advanced materials research, broadly categorized into mechanical and structural applications, device and sensor technologies, materials synthesis and characterization, and biomedical and environmental applications. Among the emerging topics, the analysis of ultrathin two-dimensional (2D) materials has attracted particular attention. Although their experimental characterization is still at an early stage, these materials are already being explored for a wide range of technological applications. Moreover, recent efforts have focused on developing nanomaterials with precisely controlled morphologies and nanoscale dimensions to achieve targeted functional properties [2]. Artificial intelligence (AI) and machine learning (ML) are now playing an increasingly important role in nanomaterials research. As noted by [3], the integration of AI and ML is expected to bridge the gap between experimental and theoretical approaches through data-driven methodologies, thereby accelerating the discovery and optimization of novel nanomaterials.

Among various classes of nanomaterials, carbon-based nanomaterials have received particular attention due to their outstanding electromechanical performance. Their exceptional mechanical strength, large surface area, and superior charge-transfer characteristics make them ideal candidates for applications in sensors, actuators, and energy devices [2]. Consequently, the present study focuses on carbon-based nanorods, which serve as essential structural elements in Nano-Electro-Mechanical Systems (NEMS) and provide a versatile platform for exploring size-dependent mechanical phenomena at the nanoscale.

The issue of static stability of an elastic rod that buckles by extension is a relatively old one, first addressed by [4]. This analysis is based on linear equations that stem from the classical Bernoulli–Euler theory of elastic rods. In the last two decades of the twentieth century, the problem regained attention through the works of [5,6]. These papers generalized the work of [4]. In [5], the classical Bernoulli–Euler theory is used for the constitutive equation, alongside nonlinear geometric relations and imperfections, to derive governing equations. These equations are then used to analyse the buckling and post-buckling of the classical Bernoulli–Euler rod. The paper [6] further generalizes [5] by considering the extensibility of the rod axis. The nonlinear analysis performed in both [5,6] indicates the presence of a supercritical bifurcation. In the context of buckling by extension of elastic rods, it is also worth mentioning [7], where an optimization problem is solved.

Our goal in this paper is to analyse the buckling and post-buckling behavior of a nanorod that can buckle by extension. This represents a generalization of the work presented in [5]. To describe the nanorod, we need an appropriate constitutive equation that takes into account nonlocal effects. It is noteworthy that the origins of nonlocal continuum mechanics theories can be traced back to the works of [8,9,10]. Since then, many theories have been developed. Among these, we highlight Eringen’s differential model [11] and Eringen’s two-phase local/nonlocal model (the local/nonlocal strain-driven model [12,13,14,15,16] on the one hand, and the nonlocal integral stress-driven model [17,18,19] and the two-phase local/nonlocal stress-driven model [20] on the other. A review of recent advances in constitutive equations of nonlocal elasticity can be found in [21,22]. A more detailed overview of stress-driven nonlocal elasticity theory is provided in [23]. It is worth noting that since [24], nonlocal theories have been frequently applied to the buckling problems of nanorods.

Considering the buckling and vibrations of nanorods using the stress-driven model as a special case of the two-phase local/nonlocal stress-driven model, several relevant papers can be mentioned. In [25] the buckling of a nanorod based on a constitutive equation that accounts only for bending effects is studied. In [26] the buckling of a nanorod whose constitutive equations incorporate both bending and shear effects is analyzed. In addition to bucking, ref. [27] also investigates the vibrations of nanorods. More recently, the buckling and bending of cracked nanobeams have been analyzed in [28,29]. All these papers reveal the presence of a stiffening effect.

In this paper, we will use the two-phase local/nonlocal stress-driven model, which generalizes the stress-driven model and ensures a well-posed problem. In addition, this model satisfies the physical requirement that the nonlocal system should be stiffer than the local one [30]. Moreover, the buckling loads predicted by this model are very similar to those obtained using strain gradient elasticity theory for small values of the length-scale parameter [25]. As we will show, this type of constitutive equation also leads to very interesting post-buckling behavior. The two-phase local/nonlocal stress-driven model has gained wide acceptance in the analysis of bending, buckling, post-buckling, vibrations, and fracture mechanics of nanorods. In particular, with regard to buckling, vibration, and dynamic analysis, we mention the papers [31,32,33,34,35,36]. On the other hand several papers related to bending include [20,37,38,39,40], while the one of the papers addressing fracture mechanics is [41].

There are not many papers addressing the post-buckling behavior of the nanorods described by the local/nonlocal stress-driven model and the two-phase local/nonlocal stress-driven model. Notable contributions include [42,43,44], which provide important insights into this relatively unexplored area.

Among the papers using the Liapunov–Schmidt method for investigating the post-buckling behavior of the nanorods we mention [45,46].

A common feature of almost all buckling problems treated within nonlocal theories is the focus on simple case studies such as simply supported beams, cantilevers, and clamped–clamped beams typically encountered in nanotechnology. As a result, we have chosen to investigate non-standard boundary conditions that arise in buckling by extension. To the best of the authors’ knowledge, the problem of buckling by extension has not yet been addressed within any nonlocal theory. We note that this issue is physically relevant, as imperfections are likely to occur during the manufacturing process of nanorods. At present, nanorods are employed across a wide range of fields in the natural sciences and engineering. In particular, carbon nanotubes have found extensive applications in industry, construction, materials science, adsorption processes, energy storage systems, sensors, actuators, semiconductor devices, water purification technologies, and various biomedical applications (see [47,48,49,50,51]). As a paper focusing on the use of nanobeams in mechanical applications (specifically for measuring the velocity of nanofluids), we mention [52].

The aim of this paper is to investigate an inextensible and unshearable cantilever nanorod to the free end of which a rigid straight rod is attached. The free end of the rigid rod is subject to a constant tensile force. We focus on the analysis of the buckling and post-buckling behavior of the nanorod through a nonlinear analysis. To this end, we employ the Liapunov–Schmidt reduction method. It is worth noting that this rigorous analytical technique has not previously been applied to problems involving the two-phase local/nonlocal stress-driven model.

The governing equations of the problem are nonlinear due to geometric nonlinearity. Depending on the parameters, linear analysis yields three distinct characteristic equations. To investigate the post-buckling behavior of the nanorod, a bifurcation equation is derived. Finally, a detailed analysis is provided on the influence of geometric and material parameters on the buckling load and the type of bifurcation. The results show that the bifurcation can be either supercritical or subcritical—an interesting and novel outcome.

## 2. Governing Equations

**Figure 1:**
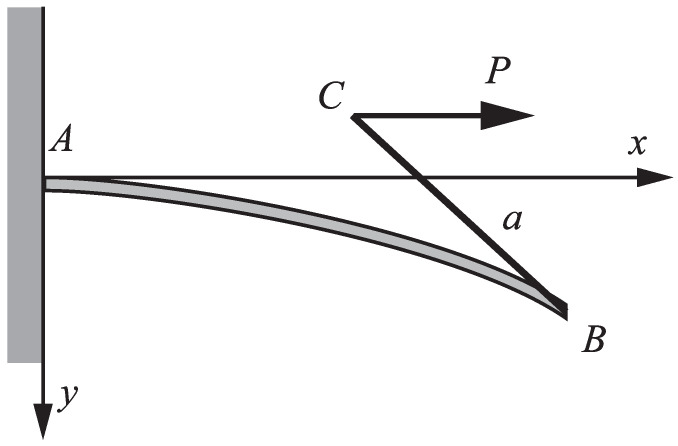
A nanorod loaded by an extensive force *P*.

Let us consider a straight, inextensible, and unshearable elastic nanorod fixed at one end *A* and free at the other end *B*. At the free end *B* a rigid straight rod BC is attached (welded) in such a way that the direction of the rigid rod and the tangent to the axis of the deformed nanorod at the end B, coincide. The lengths of the nanorod and the rigid rod are *L* and a, respectively. The arc length of the axis of the nanorod, measured from *A* is *S*. The rigid rod is loaded by a constant extensive force P which acts at the end *C*. The force acts in the direction parallel to the axis of the undeformed nanorod (Figure 1).

**Figure 2:**
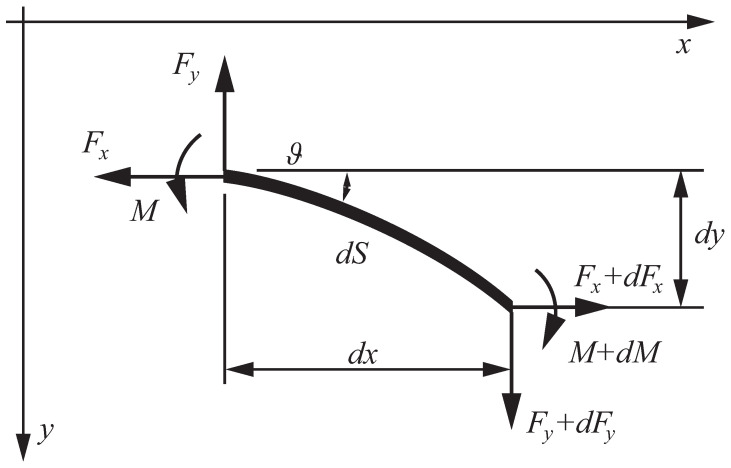
Contact forces and couples acting on an elementary part of the nanorod.

Let xAy be the coordinate system at the support *A* with the unit vectors i and j, respectively (Figure 1). Then, the equilibrium equations for an elementary segment of the nanorod read (Figure 2).

(1) $$F_{x}^{\prime }=0,\;\;\;\;\;\;\;F_{y}^{\prime }=0,\;\;\;\;\;\;\;M^{\prime }=F_{x}y^{\prime }-F_{y}x^{\prime }$$

where ${\left(\cdot \right)}^{\prime }=\frac{d(\cdot )}{dS},$ $F=F_{x}i+F_{y}j$ is the contact force, and *M* is the contact couple. The equilibrium equations are accompanied by geometrical relations (Figure 2)

(2) $$x^{\prime }=\cos\vartheta ,\;y^{\prime }=\sin\vartheta \;$$

where ϑ is the angle between the tangent to the nanorod axis and *x* axis. Next, we require a constitutive equation that takes into account nonlocal effects. As mentioned in the Introduction, we adopt the two-phase local/nonlocal stress-driven model [20]

(3) $$\kappa \left(S\right)=\zeta \frac{M(S)}{EI}+\frac{\left(1-\zeta \right)}{2lEI}\int_{0}^{L}e^{-\frac{\left|S-\eta \right|}{l}}M\left(\eta \right)d\eta$$

where l>0 is the small length-scale parameter; ζ is the phase parameter (0≤ζ≤1 ); *I* is the second moment of inertia; *E* is the modulus of elasticity; and κ is the curvature of the nanorod axis. Since for an inextensible nanorod $\kappa =\frac{d\vartheta }{dS},$ (3) can be expressed as follows [32,36,53]:

(4) $$\vartheta^{\prime }-l^{2}\vartheta^{\prime \prime \prime }=\frac{M}{EI}-\zeta \frac{M^{\prime \prime }}{EI}l^{2},$$

subject to the following:

(5) $$\begin{array}{ccc} \vartheta^{\prime \prime }\left(0\right)-\frac{1}{l}\vartheta^{\prime }\left(0\right) & = & \frac{\zeta }{EI}\left(M^{\prime }\left(0\right)-\frac{1}{l}M\left(0\right)\right),\\ \vartheta^{\prime \prime }\left(L\right)+\frac{1}{l}\vartheta^{\prime }\left(L\right) & = & \frac{\zeta }{EI}\left(M^{\prime }\left(L\right)+\frac{1}{l}M\left(L\right)\right). \end{array}$$

Since P=Pi, the equilibrium Equation (1), geometrical relations (2), and constitutive Equation (4) are accompanied by (5) and the boundary conditions (Figure 1):

(6) $$\begin{array}{ccc} F_{x}\left(L\right) & = & P,\;\;\\ F_{y}\left(L\right) & = & 0,\\ M\left(L\right) & = & Pa\sin\vartheta \left(L\right),\\ \vartheta \left(0\right) & = & 0\\ x(0) & = & 0\\ y(0) & = & 0. \end{array}$$

Now, following the idea of [46] we introduce the new variables:

(7) $$\phi =\zeta \frac{M}{EI}-\vartheta^{\prime },\;\;\;\;\;\;Q=\phi^{\prime }.$$

so that the Equations (1), (2) and (4)–(6) can be reduced to the following:

(8) $$\begin{array}{ccc} M^{\prime }-P\sin\vartheta & = & 0,\\ \vartheta^{\prime }-\zeta \frac{M}{EI}+\phi & = & 0,\\ \phi^{\prime }-Q & = & 0,\\ Q^{\prime }-\frac{\phi }{l^{2}}+\frac{\left(\zeta -1\right)M}{l^{2}EI} & = & 0, \end{array}$$

subject to the following:

(9) $$\begin{array}{ccc} M\left(L\right)-Pa\sin\vartheta \left(L\right) & = & 0,\\ \vartheta \left(0\right) & = & 0,\\ \phi (0)-lQ(0) & = & 0,\\ \phi (L)+lQ(L) & = & 0. \end{array}$$

In order to apply nonlinear analysis, we need to modify (8) and (9) to obtain linear boundary conditions. One way to achieve this is by introducing a new variable:

(10) T=Psinϑ

The use of (10) transforms the system given by (8) and (9) into the following:

(11) $$\begin{array}{ccc} M^{\prime }-T & = & 0,\\ T^{\prime }-P\left(\zeta \frac{M}{EI}-\phi \right)\cos\vartheta & = & 0,\\ \vartheta^{\prime }-\zeta \frac{M}{EI}+\phi & = & 0,\\ \phi^{\prime }-Q & = & 0,\\ Q^{\prime }-\frac{\phi }{l^{2}}+\frac{\left(\zeta -1\right)M}{l^{2}EI} & = & 0, \end{array}$$

subject to the following:

(12) $$\begin{array}{ccc} M\left(L\right)-aT\left(L\right) & = & 0,\\ T(0) & = & 0,\\ \vartheta \left(0\right) & = & 0,\\ \phi (0)-lQ(0) & = & 0,\\ \phi (L)+lQ(L) & = & 0. \end{array}$$

To facilitate the following analysis, we introduce the dimensionless quantities as follows:

(13) $$\begin{array}{ccc} t & = & \frac{S}{L},\;\bar{M}=\frac{ML}{EI},\;\bar{\phi }=\phi L,\;\bar{Q}\;=QL^{2},\\ \lambda & = & \frac{PL^{2}}{EI},\;\bar{l}=\frac{l}{L},\;\;\;\bar{T}=\frac{TL^{2}}{EI},\;\;\bar{a}=\frac{a}{L}. \end{array}$$

Now, the system (11) and (12) becomes the following:

(14) $$\begin{array}{ccc} \overset{\cdot }{\bar{M}}-\bar{T} & = & 0,\\ \overset{\cdot }{\bar{T}}-\lambda \left(\zeta \bar{M}-\;\bar{\phi }\right)\cos\vartheta & = & 0,\\ \overset{\cdot }{\vartheta }-\zeta \bar{M}+\;\bar{\phi } & = & 0,\\ \overset{\cdot }{\bar{\phi }}-\bar{Q} & = & 0,\\ \overset{\cdot }{\bar{Q}}-\frac{\bar{\phi }-\left(\zeta -1\right)\bar{M}}{\;{\bar{l}}^{2}} & = & 0, \end{array}$$

subject to the following:

(15) $$\begin{array}{ccc} \bar{M}\left(1\right)-\bar{a}\bar{T}\left(1\right) & = & 0\\ \bar{T}\left(0\right) & = & 0\\ \vartheta \left(0\right) & = & 0\\ \bar{\phi }\left(0\right)-\bar{l}\bar{Q}\left(0\right) & = & 0\\ \bar{\phi }\left(1\right)+\bar{l}\bar{Q}\left(1\right) & = & 0 \end{array}$$

where $\frac{d(\cdot )}{dt}=\overset{\cdot }{\left(\cdot \right)}.$ The system given by (14) and (15) presents the governing equations and has the trivial solution $\bar{M}=\bar{T}=\vartheta =\bar{\phi }=\bar{Q}=0$. We define *Y* as the space of vectors $y={\left(\bar{M},\bar{T},\vartheta ,\bar{\phi },\bar{Q}\right)}^{T}$ whose elements are continuously differentiable functions satisfying (15) and *Z* as the space of vectors $z={\left(z_{1},z_{2},z_{3},z_{4},z_{5}\right)}^{T}$ whose elements are continuous functions. The system described by (14) and (15) can now be formally written as an operator $\Gamma :Y\times R_{+}\to Z$ with λ being a bifurcation parameter. Note that the operator Γ(y,λ) satisfies Γ(y,λ)=−Γ(−y,λ).

## 3. Buckling Loads

In this Section, we will determine the buckling load λ as a function of the parameters $\bar{l},\zeta$ and $\bar{a}$. As a step in that direction, we first linearize (14) to obtain the following:

(16) $$\begin{array}{ccc} \overset{\cdot }{\bar{M}}-\bar{T} & = & 0,\\ \overset{\cdot }{\bar{T}}-\lambda \left(\zeta \bar{M}-\;\bar{\phi }\right) & = & 0,\\ \overset{\cdot }{\vartheta }-\zeta \bar{M}+\;\bar{\phi } & = & 0,\\ \overset{\cdot }{\bar{\phi }}-\bar{Q} & = & 0,\\ \overset{\cdot }{\bar{Q}}-\frac{\bar{\phi }-\left(\zeta -1\right)\bar{M}}{\;{\bar{l}}^{2}} & = & 0, \end{array}$$

subject to the following:

(17) $$\begin{array}{ccc} \bar{M}\left(1\right)-\bar{a}\bar{T}\left(1\right) & = & 0\\ \bar{T}\left(0\right) & = & 0\\ \vartheta \left(0\right) & = & 0\\ \bar{\phi }\left(0\right)-\bar{l}\bar{Q}\left(0\right) & = & 0\\ \bar{\phi }\left(1\right)+\bar{l}\bar{Q}\left(1\right) & = & 0 \end{array}$$

Note that the system given by (14) and (15) can be written in a compact form by defining a linear operator B(λ):Y→Z. From (16) and (17), we get the following:

(18) $${\bar{l}}^{2}\overset{\cdot \cdot \cdot \cdot }{\bar{M}}-\left(\lambda \zeta {\bar{l}}^{2}+1\right)\overset{\cdot \cdot }{\bar{M}}+\lambda \bar{M}=0,$$

subject to the following:

(19) $$\begin{array}{ccc} \overset{\cdot }{\bar{M}}\left(1\right)-\bar{a}\overset{\cdot }{\bar{M}}\left(1\right) & = & 0,\\ \overset{\cdot }{\bar{M}}\left(0\right) & = & 0,\\ \zeta \lambda \bar{M}\left(0\right)-\overset{\cdot \cdot }{\bar{M}}\left(0\right)-\zeta \lambda \bar{l}\overset{\cdot }{\bar{M}}\left(0\right)+\bar{l}\overset{\cdot \cdot \cdot }{\bar{M}}\left(0\right) & = & 0,\\ -\zeta \lambda \bar{M}\left(1\right)+\overset{\cdot \cdot }{\bar{M}}\left(1\right)-\zeta \lambda \bar{l}\overset{\cdot }{\bar{M}}\left(1\right)+\bar{l}\overset{\cdot \cdot \cdot }{\bar{M}}\left(1\right) & = & 0. \end{array}$$

We stress that for ζ=0, (18) coincides with the Equation (14) of [25]. On the other hand, for ξ=1 (18) coincides with the results of [5]. Depending on the values of parameters $\bar{l},\zeta$, $\bar{a}$ and λ the system (18), (19) has three different solutions for $\bar{M}\left(t\right)$. The detailed derivation of these solutions and the corresponding characteristic equations are presented in Appendix A. When $\bar{M}\left(t\right)$ is determined, the remaining dependent variables of the system (16), (17) are of the following form:

(20) $$\begin{array}{ccc} \bar{T} & = & \overset{\cdot }{\bar{M}}\\ \bar{\phi } & = & \zeta \bar{M}-\frac{\overset{\cdot \cdot }{\bar{M}}}{\lambda }\\ \vartheta & = & \frac{\overset{\cdot }{\bar{M}}-\overset{\cdot }{\bar{M}}\left(0\right)}{\lambda },\\ \bar{Q} & = & \zeta \overset{\cdot }{\bar{M}}-\frac{\overset{\cdot \cdot \cdot }{\bar{M}}}{\lambda }\;. \end{array}$$

Equation (20) will be used in the post-buckling analysis. The solution of linear system (16), (17) can be written in vector form as $y_{l}$ $={\left(\bar{M},\bar{T},\vartheta ,\bar{\phi },\bar{Q}\right)}^{T}$ where $\bar{M},\bar{T},\vartheta ,\bar{\phi },\bar{Q}$ are determined by (A12), (A13), (A14) and (20). We note that, in the case of the classical Bernoulli–Euler rod (ζ=1), from the system (16), (17), we get $\bar{\phi }=\bar{Q}=0$ [46]. So, in this special case, the characteristic equation becomes the following:

(21) $$\tanh\sqrt{\lambda }=\frac{1}{\bar{a}\sqrt{\lambda }}$$

which coincides with the results of [5]. In what follows, we will derive the bifurcation equation.

## 4. Post-Buckling Analysis

In order to perform the post-buckling analysis, we first define an inner product as follows:

(22) $$\langle y_{1},y_{2}\rangle =\int_{0}^{1}y_{1}^{T}y_{2}dt\;\;\;\;\;\;y_{1},y_{2}\in Y\;\;\;\;\;\;\;$$

This leads us to the adjoint $B^{*}\left(\lambda \right)$ of B(λ) which is defined as an operator that satisfies the following:

(23) $$\int_{0}^{1}{\left(B(\lambda )y\right)}^{T}qdt=\int_{0}^{1}y^{T}B^{*}\left(\lambda \right)qdt$$

where $q={\left(q_{M},q_{T},q_{\vartheta },q_{\phi },q_{Q}\right)}^{T}\in Z$. We assume that ζ≠1. Following the Liapunov–Schmidt method [54,55,56,57,58] we need the solution to $B^{*}\left(\lambda \right)q=0$ which is given in Appendix B. Due to the form of the solution (A12), (A13), (A14) and (A17) the Liapunov–Schmidt method suggests that a solution to nonlinear problems (14) and (15) takes the following form:

(24) $$y=by_{l}+v\left(by_{l},\Delta \lambda \right)$$

where *b* is a real parameter and $v(by_{l},\Delta \lambda )$ is the “extra little part” in the terminology of [55,59]. With the help of (24), the Liapunov–Schmidt method leads to the bifurcation equation:

(25) $$\int_{0}^{1}\Gamma {\left(by_{l}+v\left(by_{l},\Delta \lambda \right),\lambda_{cr}+\Delta \lambda \right)}^{T}q_{a}dt=0.$$

where $q_{a}$ is the solution to $B^{*}\left(\lambda \right)q=0$. Since Γ(y,λ)=−Γ(−y,λ) the Taylor expansion of the bifurcation equation (25) in the neighbourhood of (b,Δλ)=(0,0) leads to the following:

(26) $$b\left[c_{1}\Delta \lambda +c_{3}b^{2}+O\left(b^{4},b^{2}\Delta \lambda ,{\left(\Delta \lambda \right)}^{2}\right)\right]=0,$$

where

(27) $$\begin{array}{ccc} c_{1} & = & \frac{1-\zeta }{{\bar{l}}^{2}}\left(\bar{a}\vartheta {\left(1\right)}^{2}-\int_{0}^{1}\vartheta^{2}dt\right),\\ c_{3} & = & -\frac{1-\zeta }{{\bar{l}}^{2}}\left(\bar{a}\vartheta {\left(1\right)}^{4}-\int_{0}^{1}\vartheta^{4}dt\right). \end{array}$$

We note that the coefficients $c_{1}$ and $c_{3}$ after integration become analytical functions $c_{1}\left(\bar{l},\zeta ,\lambda_{cr}\right),\;$$c_{3}\left(\bar{l},\zeta ,\lambda_{cr}\right)$ whose explicit form will not be presented here, due to the size of the expressions. If $c_{1}\neq 0$ and $c_{3}\neq 0,$ then in the neighbourhood of (b,Δλ)=(0,0), the Equation (26) has a trivial solution (b,Δλ) = (0,Δλ) and a local one given by the following:

(28) $$\Delta \lambda =-\frac{c_{3}}{c_{1}}b^{2}+O\left(b^{4}\right).$$

This means that at $\lambda =\lambda_{cr}$ *pitchfork bifurcation* occurs. For ζ=1 the same procedure leads to the bifurcation Equation (26) where

(29) $$\begin{array}{ccc} c_{1} & = & -\int_{0}^{1}{\bar{M}}^{2}dt,\\ c_{3} & = & \frac{\lambda_{cr}}{2}\int_{0}^{1}{\bar{M}}^{2}\vartheta^{2}dt. \end{array}$$

**Figure 7:**
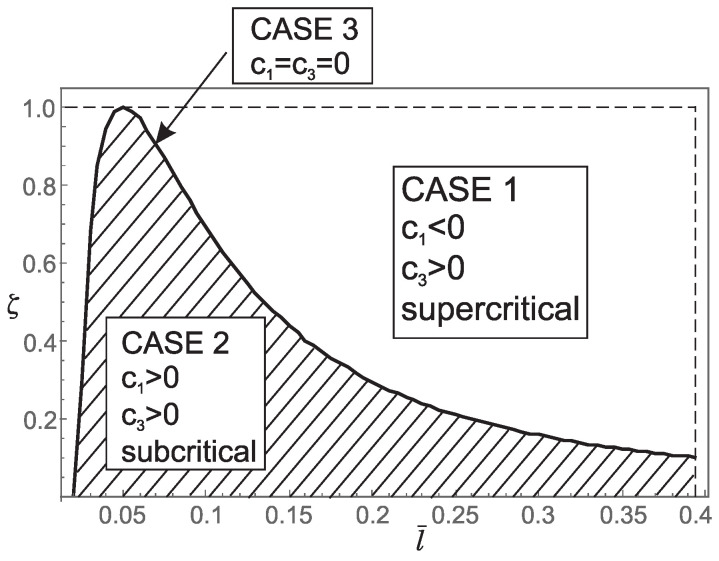
Influence of the dimensionless small length-scale parameter $\bar{l}$ and the phase parameter ζ for $\bar{a}=0.05$ on: (a) regions (Case 1, Case 2, Case 3) corresponding to the three characteristic equations that determine the buckling loads; (b) the values of coefficients $c_{1}$ and $c_{3}$ in the bifurcation equation; (c) the type of bifurcation.

**Figure 8:**
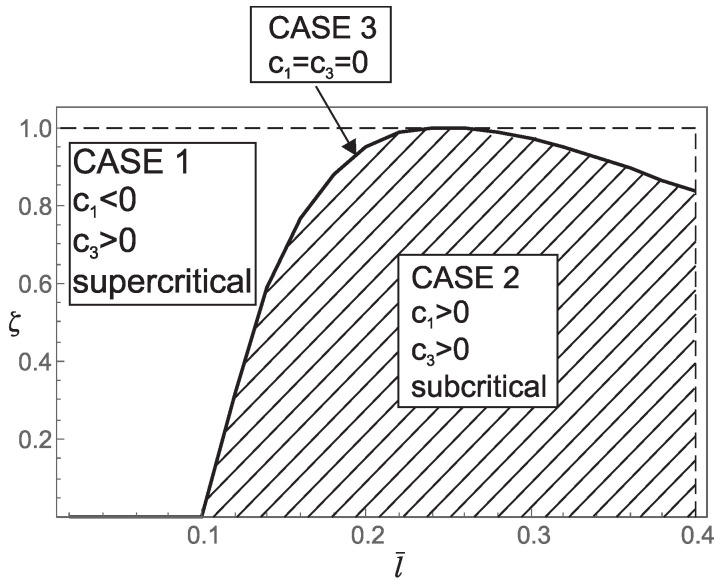
Influence of the dimensionless small length-scale parameter $\bar{l}$ and the phase parameter ζ for $\bar{a}=0.25$ on: (a) regions (Case 1, Case 2, Case 3) corresponding to the three characteristic equations that determine the buckling loads; (b) the values of coefficients $c_{1}$ and $c_{3}$ in the bifurcation equation; (c) the type of bifurcation.

**Figure 9:**
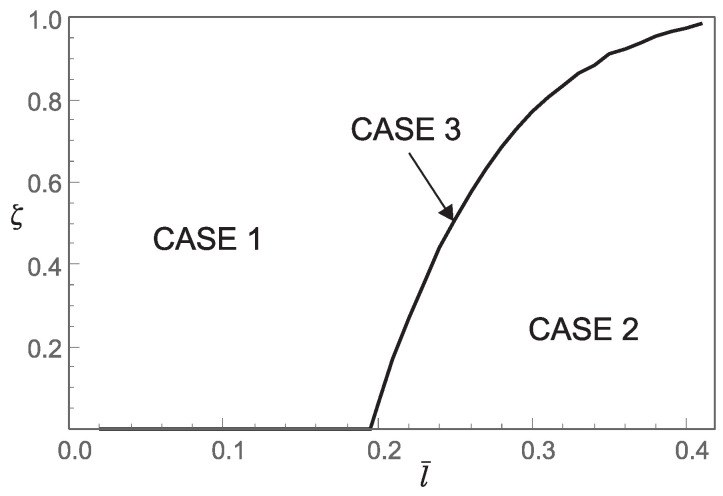
Influence of the dimensionless small length-scale parameter $\bar{l}$ and the phase parameter ζ for $\bar{a}=0.48$ on the regions (Case 1, Case 2, Case 3) corresponding to the three characteristic equations that determine the buckling loads.

**Figure 10:**
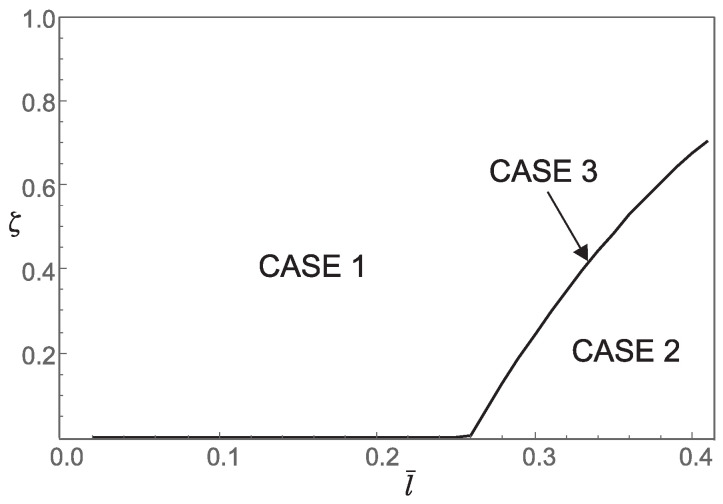
Influence of the dimensionless small length-scale parameter $\bar{l}$ and the phase parameter ζ for $\bar{a}=0.75$ on the regions (Case 1, Case 2, Case 3) corresponding to the three characteristic equations that determine the buckling loads.

**Figure 11:**
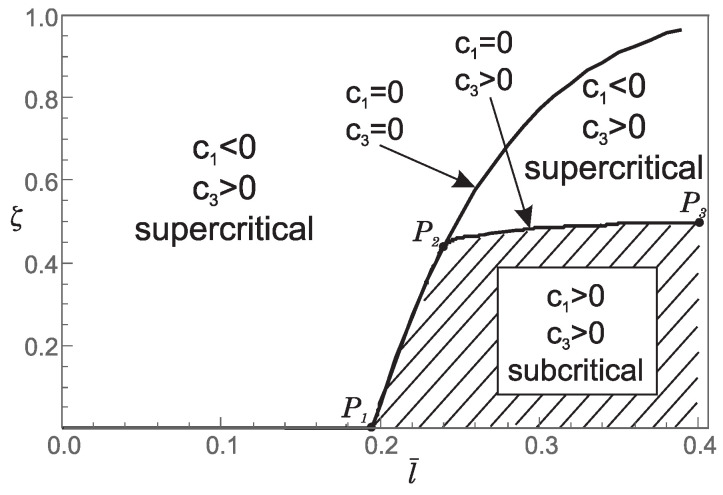
Influence of the dimensionless small length-scale parameter $\bar{l}$ and the phase parameter ζ for $\bar{a}=0.48$ on: (a) the values of the coefficients $c_{1}$ and $c_{3}$ in the bifurcation equation; (b) the type of bifurcation.

We note that the case when the conditions $c_{1}\neq 0$ and $c_{3}\neq 0$ are not satisfied will not be presented here. This analysis requires the determination of higher-order terms, which can, in principle, be obtained in analytical form. However, due to the extreme complexity of the characteristic equations (A4), (A7) and (A10), and the linear solutions (A12)–(A14) and (20), numerical calculations of these higher order terms are very difficult. Although this case could reveal some interesting results, it is not likely to occur in practice, since the ranges of the parameters ζ and $\bar{l},$ leading to this case, are relatively small (a curve) compared to the other cases (see Figures 7–11).

In the next section, we will determine the bifurcation pattern, i.e., whether the bifurcation is supercritical or subcritical.

## 5. Numerical Results and Discussion

In this section, we present numerical results regarding the buckling loads and post-buckling behavior of the nanorod.

### 5.1. Buckling Loads of Nanorod

**Figure 3:**
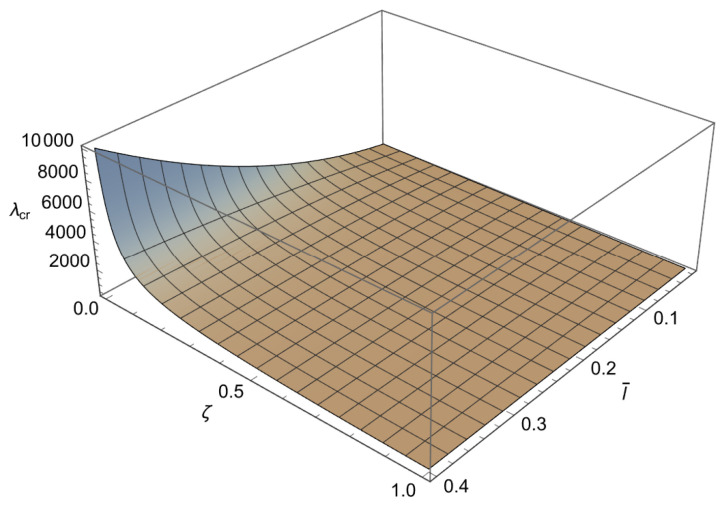
The dependence of the buckling load $\lambda_{cr}$ on the dimensionless small length-scale parameter $\bar{l}$ and the phase parameter ζ in the case of $\bar{a}=0.05$.

**Figure 4:**
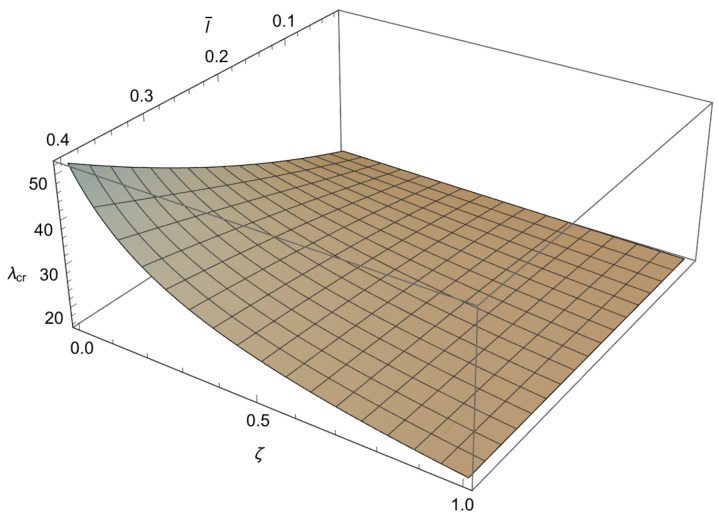
The dependence of the buckling load $\lambda_{cr}$ on the dimensionless small length-scale parameter $\bar{l}$ and the phase parameter ζ in the case of $\bar{a}=0.25$.

**Figure 5:**
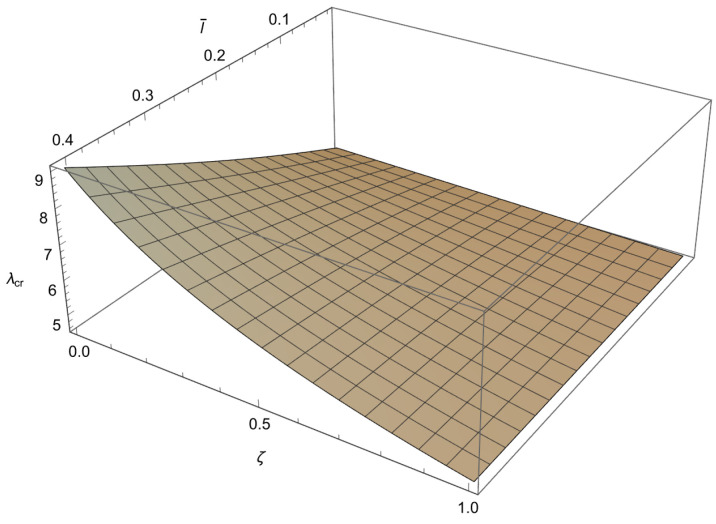
The dependence of the buckling load $\lambda_{cr}$ on the dimensionless small length-scale parameter $\bar{l}$ and the phase parameter ζ in the case of $\bar{a}=0.48$.

**Figure 6:**
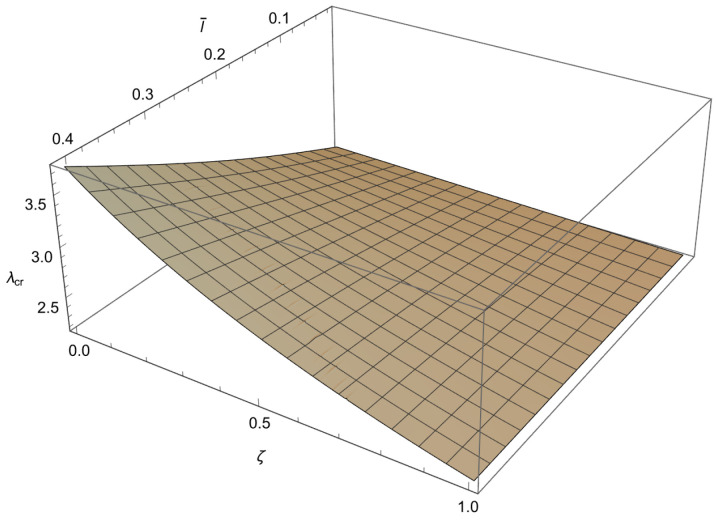
The dependence of the buckling load $\lambda_{cr}$ on the dimensionless small length scale parameter $\bar{l}$ and the phase parameter ζ in the case of $\bar{a}=0.75$.

In order to solve the characteristic equations (A4), (A7) and (A10) we will employ a numerical method. Since these characteristic equations are differentiable, the Newton–Raphson method is used, as it is fast but sensitive. This method is well known and implemented in standard numerical packages. To apply the method a mesh is formed in $\zeta \bar{l}$ plane. To achieve an accuracy of $10^{-3}$ for the buckling loads, depending on the value of $\bar{a}$, the increments for the small length-scale parameter and for the phase parameter are chosen as $\Delta \bar{l}=10^{-2}$ and $\Delta \zeta =5\times 10^{-3},$ respectively. Using the condition (A11) the characteristic equations (A4), (A7) and (A10) are solved at each node for λ, starting from the solution corresponding to the classical Bernoulli–Euler rod. In this way, the influence of the small length-scale parameter $\bar{l}$ ($0<\bar{l}\leq 0.4$) the phase parameter ζ(0≤ζ≤1) and the parameter $\bar{a}\in \left\{0.05,0.25,0.48,0.75\right\}$ on the buckling load $\lambda_{cr}$ is obtained and shown in Figure 3, Figure 4, Figure 5 and Figure 6 and Table 1, Table 2, Table 3 and Table 4.

The range of the dimensionless small length-scale parameter is adopted according to the analysis presented in [25]. In this way, the results of physical interest are adequately covered. Further increases in this parameter (≥0.4) do not lead to any new phenomenon. Namely, in nanostructures, according to [15], the value of the small length-scale parameter $\bar{l}$ typically ranges from 0.1 nm to 5 nm and depends on the specific device and material. The range of the phase parameter encompasses both the classical Bernoulli–Euler case (ζ=1) and the nonlocal integral stress-driven model (ζ=0). In engineering problems, the values of these parameters depend on the size effect, the nature of the mechanical problem, and the aspect ratio of the nanobeams [60].

From these figures, we can conclude the following:

1. For $0<\bar{l}\leq 0.4$ and $\bar{a}\in \left\{0.05,0.25,0.48,0.75\right\}$ a decrease in ζ causes an increase in $\lambda_{cr}.$

2. For 0≤ζ≤1 and $\bar{a}\in \left\{0.05,0.25,0.48,0.75\right\}$ an increase in $\bar{l}$ leads to an increase in $\lambda_{cr}.$

3. For $0<\bar{l}\leq 0.4$ and 0≤ζ≤1 an increase in $\bar{a}$ causes a decrease in $\lambda_{cr}.$

4. The influence of the small length-scale parameter $\bar{l}$ and the phase parameter ζ on the critical buckling load $\lambda_{cr}$ diminishes as the parameter $\bar{a}$ increases.

5. In the special case when ζ approaches 1 or $\bar{l}$ tends to 0 the buckling load $\lambda_{cr}$ tends to the value corresponding to the classical Bernoulli–Euler theory as obtained from (21). In particular, for $\bar{a}=\left\{0.05,0.25,0.48,0.75\right\}$ the critical buckling loads of the classical Bernoulli-Euler theory are $\lambda_{cr}=\left\{400,16.0213,4.5865,2.1882\right\}$, respectively.

Thus, from points 1 and 2, we conclude that the *stiffening effect* is present, as expected. However, the influence of nonlocality significantly decreases as $\bar{a}$ increases. As shown in Section 3, there are three different characteristic equations (Case 1, Case 2 and Case 3) depending on the value of $k^{4}-d^{4}.$ For $\bar{a}\in \left\{0.05,0.25,0.48,0.75\right\},$ using (A11), we can determine Case 3, which is the boundary between Case 1 and Case 2. This boundary is presented in the $\bar{l}\zeta$ diagrams shown in Figure 7, Figure 8, Figure 9 and Figure 10. These figures show that for $(\bar{l},\zeta )\in \left(0,0.4\right]$ ×[0,1], an increase in $\bar{a}$ shifts the region corresponding to Case 2 to the right.

### 5.2. Post-Buckling Behavior of Nanorod

Although the occurrence of the three cases in the buckling analysis is interesting from a mathematical point of view, these cases also play an important role in the post-buckling behavior of the nanorod. To show this, we recall that a pitchfork bifurcation can be supercritical if $-\frac{c_{3}}{c_{1}}>0$ and subcritical if $-\frac{c_{3}}{c_{1}}<0$. In the special case of the classical Bernoulli-Euler theory, (29) indicates that the bifurcation is supercritical, which is in agreement with [5]. For the general case, after performing the analytical integration, the coefficients $c_{1}$ and $c_{3}$ become analytical functions that are not suitable for presentation due to their extreme length. Numerical calculation of these functions leads to the following conclusions:

1. For $\bar{a}\in \left\{0.05,0.25\right\}$ and $\left(\bar{l},\zeta \right)\in \left(0,0.4\right]\times \left[0,1\right]$ the coefficients $c_{1}$ and $c_{3}$ take the following values:

(30) $$\begin{array}{ccc} c_{1} & < & 0\;\;\mathrm{and}\;c_{3}>0\;\mathrm{if}\;\;\;\;k^{4}-d^{4}>0\\ c_{1} & = & 0\;\;\mathrm{and}\;c_{3}=0\;\mathrm{if}\;\;\;\;k^{4}-d^{4}=0\\ c_{1} & > & 0\;\;\mathrm{and}\;c_{3}>0\;\mathrm{if}\;\;\;\;k^{4}-d^{4}<0 \end{array}$$

This implies that for $\left(\bar{l},\zeta \right)$ belonging to the region corresponding to Case 1 in Figure 7 and Figure 8, supercritical bifurcation occurs. Conversely, for $\left(\bar{l},\zeta \right)$ belonging to the region corresponding to Case 2 in Figure 7 and Figure 8, subcritical bifurcation is observed. On the boundary between Case 1 and Case 2 (Case 3, $k^{4}-d^{4}=0$) we have $c_{1}=c_{3}=0,$ and thus additional analysis is needed to determine the bifurcation pattern. Therefore, the boundary between Case 1 and Case 2 (Case 3, $k^{4}-d^{4}=0$) separates supercritical from subcritical bifurcation. It is noteworthy that subcritical bifurcation in real-world constructions is highly undesirable due to its potential for sudden and unstable structural behavior.

2. For $\bar{a}=0.48$ and $\left(\bar{l},\zeta \right)\in \left(0,0.4\right]\times \left[0,1\right]$ the values of the coefficients $c_{1}$ and $c_{3},$ as well as the bifurcation pattern, are shown in Figure 11. This figure shows that both supercritical and subcritical bifurcations occur. However, from Figure 11, it can be concluded that supercritical and subcritical bifurcations are only partially separated by the boundary between Case 1 and Case 2 (part $P_{1}P_{2}$), while the remaining boundary follows from $c_{1}=0$ and $c_{3}>0$ (part $P_{2}P_{3}$). Moreover, on the boundary between Case 1 and Case 2, we again have $c_{1}=c_{3}=0,$ which means that the bifurcation pattern cannot be determined without additional analysis.

3. For $\bar{a}=0.75$ and $\left(\bar{l},\zeta \right)\in \left(0,0.4\right]\times \left[0,1\right]$ numerical calculations show that $c_{1}<0$ and $c_{3}>0.$ This means that only a supercritical bifurcation occurs in this case.

These results suggest that for smaller values of the parameter $\bar{a}$ nonlocal effect can lead to both super and subcritical bifurcation. On the other hand, for larger values of the parameter $\bar{a}$ the nonlocal effect does not lead to subcritical bifurcation, meaning that only supercritical bifurcation occurs as in the classical Bernoulli-Euler theory. Based on these observations, we conclude that for larger values of $\bar{a}$ the post-buckling behavior of the nanorod is qualitatively the same as that of the rod described by the classical Bernoulli–Euler theory. It is also interesting to note that, in all cases under consideration, the change in bifurcation type occurs when $c_{1}$ changes sign.

In concluding this section, we emphasize that the validity of the theoretical results must be verified before they are applied to real engineering problems. This validation is typically carried out through experimental studies or molecular dynamics (MD) simulations. Experimental methods face inherent challenges when dealing with measurements at the nanoscale. However, they remain valuable for determining material properties. In the present problem, experimental validation of the theoretical results would likely be difficult due to the relatively complex boundary conditions. On the other hand, MD simulations are time-consuming and computationally demanding. Nevertheless, recent advances in computer science, particularly in ML and AI, suggest that MD simulations will become increasingly widespread in the future for studying and solving realistic nanoscale problems. In the analysis of nanorods, due to their tiny nature, MD plays a crucial role in predicting their mechanical behavior and properties [60]. It is worth noting that the constitutive form of the two-phase local/nonlocal stress-driven model is particularly well-suited for MD-based analysis. For the present problem, MD simulations could be employed to calculate the buckling loads. By comparing these results with theoretical predictions, one could get an approximate value of the phase parameter ζ for a given nanorod material, thus providing design guidance for nanodevice fabrication. However, as reported by [61], a complete agreement between theoretical and MD results may not be achievable across all parameter ranges. Finally, we note that the validation of the buckling loads of compressed nanobeams for the nonlocal integral stress-driven model (a special case of the model adopted in this work) was performed in [25] by comparing the results with those obtained from the theory of strain gradient elasticity.

## 6. Conclusions

This paper analyzes a nano-cantilever that buckles by extension. The constitutive equation, which takes into account nonlocal effects, is based on the two-phase local/nonlocal stress-driven model. The main results are summarized as follows:

1. The nonlinear differential governing equations of the nanorod, (14) and (15), based on the two-phase local/nonlocal stress-driven model, are derived in the form that is adequate for nonlinear analysis since the nonlinear boundary conditions are eliminated.

2. Three characteristic equations are derived (depending on the values of parameters), which is a compelling result. For the values of the parameters under consideration, the *stiffening effect* is observed. Namely, the presence of a nonlocal effect increases the buckling loads. At the same time, the influence oft he parameter $\bar{a}$ (the dimensionless length of the rigid rod) qualitatively stays the same as in the classical Bernoulli-Euler theory, meaning that an increase in $\bar{a}$ leads to a decrease in the buckling load $\lambda_{cr}$. If nonlocal effects disappear, the buckling loads are in agreement with the results of the classical Bernoulli-Euler theory.

3. A rigorous mathematical approach that uses the Liapunov–Schmidt method shows that the buckling loads determine the bifurcation points.

4. The post-buckling behavior of the nanorod is determined by the bifurcation Equation (26), which reveals that the bifurcation can be both supercritical and subcritical. In particular, for smaller values of the parameter $\bar{a}$, the influence of the nonlocal effect causes both *supercritical and subcritical* bifurcations, while for larger values of $\bar{a}$, the nonlocal effect leads only to supercritical bifurcation. The appearance of subcritical bifurcation is a new result that was not observed in the classical Bernoulli-Euler theory. Namely, to the best of the authors’ knowledge, papers discussing post-buckling behavior usually inherit entirely the behavior corresponding to the classical Bernoulli-Euler theory. Since this is not the case here, we consider this a significant result.

5. As in previous papers using the two-phase local/nonlocal stress-driven model, the presence of both the small length-scale parameter and the phase parameter allows us, for a fixed small length-scale parameter, to calibrate the phase parameter in order to more realistically describe the properties of the nanomaterial.

## Figures and Tables

**Table 1:** Numerical values of the buckling load $\lambda_{cr}$ for $\bar{a}=0.05$ and several values of the dimensionless small-length scale parameter $\bar{l}$ and the phase parameter ζ.

ζ/$\bar{l}$	$\frac{1}{50}$	$\frac{1}{10}$	$\frac{1}{5}$	$\frac{3}{10}$	$\frac{2}{5}$
**1**	400	400	400	400	400
$\frac{1}{2}$	468.9498	611.1456	678.8897	711.1087	729.8253
$\frac{1}{200}$	574.6217	1568.7782	3398.9511	5730.7799	8392.8216

**Table 2:** Numerical values of the buckling load $\lambda_{cr}$ for $\bar{a}=0.25$ and several values of the dimensionless small-length scale parameter $\bar{l}$ and the phase parameter ζ.

ζ/$\bar{l}$	$\frac{1}{50}$	$\frac{1}{10}$	$\frac{1}{5}$	$\frac{3}{10}$	$\frac{2}{5}$
**1**	16.0213	16.0213	16.0213	16.0213	16.0213
$\frac{1}{2}$	16.6414	18.7717	20.8000	22.3237	23.4916
$\frac{1}{200}$	17.3176	22.9900	31.1948	40.6795	51.4316

**Table 3:** Numerical values of the buckling load $\lambda_{cr}$ for $\bar{a}=0.48$ and several values of the dimensionless small-length scale parameter $\bar{l}$ and the phase parameter ζ.

ζ/$\bar{l}$	$\frac{1}{50}$	$\frac{1}{10}$	$\frac{1}{5}$	$\frac{3}{10}$	$\frac{2}{5}$
**1**	4.5865	4.5865	4.5865	4.5865	4.5865
$\frac{1}{2}$	4.6799	5.021	5.3904	5.7124	5.9923
$\frac{1}{200}$	4.7771	5.5625	6.6052	7.7228	8.8971

**Table 4:** Numerical values of the buckling load $\lambda_{cr}$ for $\bar{a}=0.75$ and several values of the dimensionless small-length scale parameter $\bar{l}$ and the phase parameter ζ.

ζ/$\bar{l}$	$\frac{1}{50}$	$\frac{1}{10}$	$\frac{1}{5}$	$\frac{3}{10}$	$\frac{2}{5}$
**1**	2.1882	2.1882	2.1882	2.1882	2.1882
$\frac{1}{2}$	2.2205	2.3427	2.4866	2.6225	2.7472
$\frac{1}{200}$	2.2534	2.5215	2.8827	3.2783	3.6984

## Data Availability

Data are contained within the article.

## Appendix A

In this appendix, we report the mathematical steps taken to solve (19). To obtain these solutions, we follow the classical approach [62] by trying a function of the form:

$$\bar{M}\left(t\right)=e^{rt}$$

which, when substituted into (18) yields the auxiliary equation as follows:

(A1) $${\bar{l}}^{2}r^{4}-\left(\lambda \zeta {\bar{l}}^{2}+1\right)r^{2}+\lambda =0.$$

Next, if we define two quantities as follows:

$$k=\sqrt{\frac{1+\lambda \zeta {\bar{l}}^{2}}{4{\bar{l}}^{2}}},\;\;\;\;\;d=\sqrt[{4}]{\frac{\lambda }{4{\bar{l}}^{2}}},$$

the roots of (A1) depend on the value of the expression $k^{4}-d^{4}$ [58,63]. Namely, we will analyze the following three cases:

**Case 1** $\left(k^{4}-d^{4}>0\right)$

In this case, we assume that $k^{4}-d^{4}>0$, so that the roots of (A1) are real and distinct:

$$r_{1,2,3,4}=\pm \left(\sqrt{k^{2}+d^{2}}\pm \sqrt{k^{2}-d^{2}}\right).$$

By introducing the quantities:

(A2) $$\alpha_{1,2}=\sqrt{k^{2}+d^{2}}\pm \sqrt{k^{2}-d^{2}},$$

the general solution to (18) becomes the following:

(A3) $$\bar{M}\left(t\right)=C_{11}\sinh\alpha_{1}t+C_{12}\cosh\alpha_{1}t+C_{13}\sinh\alpha_{2}t+C_{14}\cosh\alpha_{2}t,$$

where $C_{1i}\;i=1,\ldots ,4$ are arbitrary real constants. Substituting (A3) into the boundary conditions (19), we get the characteristic equation

(A4) $$\begin{array}{c} \alpha_{2}\cosh\left(\alpha_{2}\right)\left\{2\alpha_{1}\cosh\left(\alpha_{1}\right)\left[\alpha_{2}^{2}\alpha_{1}^{2}\bar{a}-\left(\alpha_{1}^{2}+\alpha_{2}^{2}\right)\zeta \lambda \left(\bar{a}+\bar{l}\right)+\zeta^{2}\lambda^{2}\left(\bar{a}+\bar{l}\right)+\alpha_{1}^{4}\bar{l}-\alpha_{2}^{2}\alpha_{1}^{2}\bar{l}+\alpha_{2}^{4}\bar{l}\right]+\right.\\ \left.+\left(\alpha_{1}-\alpha_{2}\right)\left(\alpha_{1}+\alpha_{2}\right)\sinh\left(\alpha_{1}\right)\left[\alpha_{1}^{2}\left(\bar{l}\left(2\alpha_{2}^{2}\bar{a}-\zeta \lambda \left(2\bar{a}+\bar{l}\right)+\alpha_{1}^{2}\bar{l}\right)+1\right)-\zeta \lambda \right]\right\}\\ -\sinh\left(\alpha_{2}\right)\left\{\sinh\left(\alpha_{1}\right)\left\{\left(\alpha_{1}^{2}+\alpha_{2}^{2}\right)\zeta^{2}\lambda^{2}\left(\bar{a}+\bar{l}\right)-\zeta \lambda \left[4\alpha_{1}^{2}\alpha_{2}^{2}\bar{a}+{\left(\alpha_{1}^{2}+\alpha_{2}^{2}\right)}^{2}\bar{l}\right]\right.\right.\\ \left.+\alpha_{1}^{2}\alpha_{2}^{2}\left[\alpha_{2}^{4}\bar{a}{\bar{l}}^{2}+\alpha_{2}^{2}\left(-2\alpha_{1}^{2}\bar{a}{\bar{l}}^{2}+\bar{a}+\bar{l}\right)+\alpha_{1}^{2}\left(\alpha_{1}^{2}\bar{a}{\bar{l}}^{2}+\bar{a}+\bar{l}\right)\right]\right\}\\ \left.+\alpha_{1}\left(\alpha_{1}-\alpha_{2}\right)\left(\alpha_{1}+\alpha_{2}\right)\cosh\left(\alpha_{1}\right)\left[\alpha_{2}^{2}\left[\bar{l}\left(2\alpha_{1}^{2}\bar{a}-\zeta \lambda \left(2\bar{a}+\bar{l}\right)+\alpha_{2}^{2}\bar{l}\right)+1\right]-\zeta \lambda \right]\right\}\\ -2\alpha_{1}\alpha_{2}\left(\bar{a}+\bar{l}\right)\left(\alpha_{1}^{2}-\zeta \lambda \right)\left(\alpha_{2}^{2}-\zeta \lambda \right)=0 \end{array}$$

This equation ensures the existence of a solution to the linear system (18) and (19).

**Case 2** $\left(k^{4}-d^{4}<0\right)$

Now, we assume that $k^{4}-d^{4}<0$. The roots of (A1) are two complex conjugate pairs of the form:

$$r_{1,2,3,4}=\pm \left(\sqrt{k^{2}+d^{2}}\pm i\sqrt{d^{2}-k^{2}}\right)$$

Defining the following quantities:

(A5) $$\alpha_{3}=\sqrt{k^{2}+d^{2}},\;\;\;\;\alpha_{4}=\sqrt{d^{2}-k^{2}}$$

the general solution to (18) reads as follows:

(A6) $$\bar{M}\left(t\right)=C_{21}\sinh\alpha_{3}t\sin\alpha_{4}t+C_{22}\sinh\alpha_{3}t\cos\alpha_{4}t+C_{23}\cosh\alpha_{3}t\sin\alpha_{4}t+C_{24}\cosh\alpha_{3}t\cos\alpha_{4}t$$

where $C_{2i}\;i=1,\ldots ,4$ are arbitrary real constants. The substitution of this solution into (19) yields the characteristic equation:

(A7) $$\begin{array}{c} \left(\alpha_{3}^{2}+\alpha_{4}^{2}\right)\left(\bar{a}+\bar{l}\right)\left[2\alpha_{3}^{2}\left(\alpha_{4}^{2}-\zeta \lambda \right)+{\left(\alpha_{4}^{2}+\zeta \lambda \right)}^{2}+\alpha_{3}^{4}\right]-2\alpha_{3}\alpha_{4}^{2}\sinh\left(2\alpha_{3}\right)\left\{\zeta \lambda \left[\left(\left(\alpha_{3}^{2}\right.+\alpha_{4}^{2}\right)\right]\right.\\ \left.\left.\alpha_{4}^{2}\right)\bar{l}\left(2\bar{a}+\bar{l}\right)-1\right]\left.+\left(\alpha_{3}^{2}+\alpha_{4}^{2}\right)\left[\alpha_{3}^{2}\bar{l}\left(2\bar{a}-3\bar{l}\right)+\alpha_{4}^{2}\bar{l}\left(2\bar{a}+\bar{l}\right)-1\right]\right\}-\alpha_{4}^{2}\cosh\left(2\alpha_{3}\right)\left\{2\zeta \lambda \right.\\ \left.\left[\alpha_{3}^{2}\left(\bar{a}-\bar{l}\right)+\alpha_{4}^{2}\left(\bar{a}+\bar{l}\right)\right]+\left(\alpha_{3}^{2}+\alpha_{4}^{2}\right)\left[\left(\alpha_{3}^{2}+\alpha_{4}^{2}\right)\bar{a}\left(4\alpha_{3}^{2}{\bar{l}}^{2}+1\right)+\left(\alpha_{4}^{2}-7\alpha_{3}^{2}\right)\bar{l}\right]+\zeta^{2}\lambda^{2}\left(\bar{a}+\bar{l}\right)\right\}\\ +\alpha_{3}^{2}\left\{\cos\left(2\alpha_{4}\right)\left\{2\zeta \lambda \left[\alpha_{3}^{2}\left(\bar{a}+\bar{l}\right)+\alpha_{4}^{2}\left(\bar{a}-\bar{l}\right)\right]+\left(\alpha_{3}^{2}+\alpha_{4}^{2}\right)\left[\left(\alpha_{3}^{2}+\alpha_{4}^{2}\right)\bar{a}\left(4\alpha_{4}^{2}{\bar{l}}^{2}-1\right)-\left(\alpha_{3}^{2}-7\alpha_{4}^{2}\right)\bar{l}\right]\right.\right.\\ \left.-\zeta^{2}\lambda^{2}\left(\bar{a}+\bar{l}\right)+2\alpha_{4}\sin\left(2\alpha_{4}\right)\left\{\left(\alpha_{3}^{2}+\alpha_{4}^{2}\right)\left[\alpha_{3}^{2}\bar{l}\left(2\bar{a}+\bar{l}\right)+\alpha_{4}^{2}\bar{l}\left(2\bar{a}-3\bar{l}\right)+1\right]\right.\right.\\ \left.\left.-\zeta \lambda \left[\left(\alpha_{3}^{2}+\alpha_{4}^{2}\right)\bar{l}\left(2\bar{a}+\bar{l}\right)+1\right]\right\}\right\}=0 \end{array}$$

**Case 3** $\left(k^{4}-d^{4}=0\right)$

Taking into account $k^{4}-d^{4}=0,$ the roots of (A1) are as follows:

$$r_{1,2}=\sqrt{2}k,\;\;\;r_{3,4}=-\sqrt{2}k.$$

so there are two repeated real roots. Introducing the quantity:

(A8) $$\alpha_{5}=\sqrt{2}k$$

the general solution to (18) is as follows:

(A9) $$\bar{M}\left(t\right)=C_{31}\sinh\alpha_{5}t+C_{32}t\sinh\alpha_{5}t+C_{33}\cosh\alpha_{5}t+C_{34}t\cosh\alpha_{5}t,$$

where $C_{3i}\;i=1,\ldots ,4$ are arbitrary real constants. As in the previous two cases, the solution (A9) together with the boundary conditions (19) leads to the characteristic equation:

(A10) $$\begin{array}{c} 2\alpha_{5}^{6}\left[2\left(\bar{a}+1\right){\bar{l}}^{2}+4\bar{a}\bar{l}+\bar{a}+\bar{l}\right]+\alpha_{5}^{4}\left[-4\lambda \zeta \left(2\bar{a}\bar{l}+\bar{a}+{\bar{l}}^{2}+\bar{l}\right)+\bar{a}+9\bar{l}+4\right]\\ -\cosh\left(2\alpha_{5}\right)\left[\alpha_{5}^{4}\left(4\alpha_{5}^{2}\bar{a}{\bar{l}}^{2}+\bar{a}-7\bar{l}\right)+2\alpha_{5}^{2}\lambda \zeta \left(\bar{a}-\bar{l}\right)+\lambda^{2}\zeta^{2}\left(\bar{a}+\bar{l}\right)\right]+2\alpha_{5}^{2}\lambda \zeta \left[\lambda \zeta (b+l)\right.\\ \left.+b-l-2\right]+2\alpha_{5}\sinh\left(2\alpha_{5}\right)\left[\alpha_{5}^{4}\bar{l}\left(3\bar{l}-2\bar{a}\right)+\alpha_{5}^{2}\left(1-\lambda \bar{l}\zeta \left(2\bar{a}+\bar{l}\right)\right)+\lambda \zeta \right]+\lambda^{2}\zeta^{2}\left(\bar{a}+\bar{l}\right)=0 \end{array}$$

It is worth noting that the condition determining which case should be used can be stated as follows:

(A11) $$\begin{array}{ccc} k^{4}-d^{4} & > & 0\;\;\;\;\Leftrightarrow \;\sqrt{\lambda }<\;\frac{1-\sqrt{1-\zeta }}{\zeta \bar{l}}\;\;\;or\;\sqrt{\lambda }>\;\frac{1+\sqrt{1-\zeta }}{\zeta \bar{l}}\\ k^{4}-d^{4} & < & 0\;\;\;\;\Leftrightarrow \;\;\frac{1-\sqrt{1-\zeta }}{\zeta \bar{l}}<\;\sqrt{\lambda }<\;\frac{1+\sqrt{1-\zeta }}{\zeta \bar{l}}\\ k^{4}-d^{4} & = & 0\;\;\;\;\Leftrightarrow \;\sqrt{\lambda }=\frac{1-\sqrt{1-\zeta }}{\zeta \bar{l}}\;\;\;or\;\;\;\sqrt{\lambda }=\frac{1+\sqrt{1-\zeta }}{\zeta \bar{l}} \end{array}$$

Depending on the given values of $\zeta ,\bar{a}$ and $\bar{l}$ the appropriate characteristic equation (one of (A4), (A7) or (A10)), determines the values of λ. Similar to [5,6], numerical analysis indicates that for given $\zeta ,\bar{a}$ and $\bar{l}$ there is only one positive value of λ, which we will refer to as the buckling load and denote by $\lambda_{cr}$.

In **Case 1**, the solution to the linear system (18), (19), corresponding to $\lambda =\lambda_{cr}$, is given by the following:

(A12) $$\begin{array}{ccc} \bar{M}\left(t\right) & = & G_{1}\left\{\left[\alpha_{2}\sinh\left(\alpha_{1}t\right)-\alpha_{1}\sinh\left(\alpha_{2}t\right)\right]\left[\left(\zeta \lambda -\alpha_{2}^{2}\right)\left(\cosh\alpha_{1}-\alpha_{1}\bar{a}\sinh\alpha_{1}\right)\right.\right.\\ & & \left.-\left(\zeta \lambda -\alpha_{1}^{2}\right)\left(\cosh\alpha_{2}-\alpha_{2}\bar{a}\sinh\alpha_{2}\right)\right]+\cosh\left(\alpha_{2}t\right)\left[\alpha_{1}\left(\alpha_{1}^{2}-\zeta \lambda \right)\left(\sinh\alpha_{2}\right.\right.\\ & & \left.-\alpha_{2}\bar{a}\cosh\alpha_{2}\right)+\alpha_{2}\sinh\alpha_{1}\left[\zeta \lambda -\alpha_{1}^{2}\left(1+\left(\alpha_{2}^{2}-\alpha_{1}^{2}\right)\bar{a}\bar{l}\right)\right]\\ & & \left.-\alpha_{1}\alpha_{2}\cosh\alpha_{1}\left[\bar{a}\left(\zeta \lambda -\alpha_{1}^{2}\right)-\left(\alpha_{2}^{2}-\alpha_{1}^{2}\right)\bar{l}\right]\right]+\cosh\left(\alpha_{1}t\right)\left[\alpha_{2}\left(\alpha_{2}^{2}\right.\right.\\ & & \left.-\zeta \lambda \right)\left(\sinh\alpha_{1}-\alpha_{1}\bar{a}\cosh\alpha_{1}\right)++\alpha_{1}\sinh\alpha_{2}\left[\zeta \lambda -\alpha_{2}^{2}\left(1-\left(\alpha_{2}^{2}-\alpha_{1}^{2}\right)\bar{a}\bar{l}\right)\right]\\ & & \left.\left.-\alpha_{1}\alpha_{2}\cosh\alpha_{2}\left[\bar{a}\left(\zeta \lambda -\alpha_{2}^{2}\right)+\left(\alpha_{2}^{2}-\alpha_{1}^{2}\right)\bar{l}\right]\right.\right\} \end{array}$$

where $G_{1}$ is an arbitrary constant.

In **Case 2**, the solution to the linear system (18), (19), corresponding to $\lambda =\lambda_{cr}$, reads as follows:

(A13) $$\begin{array}{ccc} \bar{M}\left(t\right) & = & G_{2}\left\{\alpha_{3}\cosh\alpha_{3}\left\{\alpha_{3}\cosh\left(\alpha_{3}t\right)\left[2\alpha_{4}\sin\alpha_{4}\cos\left(\alpha_{4}t\right)\left(\bar{a}\bar{l}\left({\alpha_{3}}^{2}+{\alpha_{4}}^{2}\right)-1\right)\right.\right.\right.\\ & & \left.+\sin\left(\alpha_{4}t\right)\left(2\alpha_{4}\cos\alpha_{4}+\bar{a}\left({\alpha_{3}}^{2}+{\alpha_{4}}^{2}-\lambda \zeta \right)\sin\alpha_{4}\right)\right]\\ & & \left.+\sinh\left(\alpha_{3}t\right)\left[\sin\left(\alpha_{4}t\right)\left(\sin\alpha_{4}\left({\alpha_{3}}^{2}-{\alpha_{4}}^{2}+2{\alpha_{3}}^{2}{\alpha_{4}}^{2}\bar{a}\bar{l}+2{\alpha_{4}}^{4}\bar{a}\bar{l}-\lambda \zeta \right)\right.\right.\right.\\ & & \left.+2\alpha_{4}\bar{l}\left({\alpha_{3}}^{2}+{\alpha_{4}}^{2}\right)\cos\alpha_{4}\right)-\alpha_{4}\cos\left(\alpha_{4}t\right)\left(2\alpha_{4}\cos\alpha_{4}\right.\\ & & \left.\left.\left.+\bar{a}\left({\alpha_{3}}^{2}+{\alpha_{4}}^{2}-\lambda \zeta \right)\sin\alpha_{4}\right)\right]\right\}+\sinh\alpha_{3}\left\{\sinh\left(\alpha_{3}t\right)\left[\alpha_{4}\cos\alpha_{4}\left(\sin(\alpha_{4}t)\right.\right.\right.\\ & & \left({\alpha_{4}}^{2}-{\alpha_{3}}^{2}\left(2\bar{a}\bar{l}\left({\alpha_{3}}^{2}+{\alpha_{4}}^{2}\right)+1\right)+\lambda \zeta \right)\left.+\alpha_{4}\bar{a}\cos\left(\alpha_{4}t\right)\left({\alpha_{3}}^{2}+{\alpha_{4}}^{2}+\lambda \zeta \right)\right)\\ & & \left.+\sin\alpha_{4}\left(-{\alpha_{3}}^{2}+{\alpha_{4}}^{2}+\lambda \zeta \right)\left(\bar{a}\left({\alpha_{3}}^{2}+{\alpha_{4}}^{2}\right)\sin\left(\alpha_{4}t\right)-\alpha_{4}\cos\left(\alpha_{4}t\right)\right)\right]\\ & & \left.\left.+\alpha_{3}\cosh\left(\alpha_{3}t\right)\left[\sin\alpha_{4}\left(2\alpha_{4}\left({\alpha_{3}}^{2}+{\alpha_{4}}^{2}\right)\left(\bar{a}-\bar{l}\right)\cos\left(\alpha_{4}t\right)\right.\right.\right.\right.\\ & & \left.+\sin\left(\alpha_{4}t\right)\left(-{\alpha_{3}}^{2}+{\alpha_{4}}^{2}+\lambda \zeta \right)\right)+\alpha_{4}\cos\alpha_{4}\left(2\alpha_{4}\cos\left(\alpha_{4}t\right)\left(\bar{a}\bar{l}\left({\alpha_{3}}^{2}+{\alpha_{4}}^{2}\right)+1\right)\right.\\ & & \left.\left.\left.\left.-\bar{a}\sin\left(\alpha_{4}t\right)\left({\alpha_{3}}^{2}+{\alpha_{4}}^{2}+\lambda \zeta \right)\right)\right]\right\}\right\}. \end{array}$$

where $G_{2}$ is an arbitrary constant.

In **Case 3** the solution to the linear system (18), (19), corresponding to $\lambda =\lambda_{cr}$, is of the form

(A14) $$\begin{array}{ccc} \bar{M}\left(t\right) & = & G_{3}\left\{\alpha_{5}\cosh\left(\alpha_{5}t\right)\left[\sinh\alpha_{5}\left[\alpha_{5}^{2}\left(2\left(\left(\bar{a}-1\right)\bar{l}+\bar{a}\right)-\left(\bar{a}+1\right)t\right)-\left(\bar{a}-1\right)\lambda \zeta t+2\right.\right.\right.\\ & & \left.+\alpha_{5}\cosh\alpha_{5}\left[2\alpha_{5}^{2}\bar{a}\bar{l}+t\left(\alpha_{5}^{2}\bar{a}-\bar{a}\lambda \zeta +2\right)-2\right]\right]\\ & & \left.+\sinh\left(\alpha_{5}t\right)\left[\alpha_{5}\cosh\alpha_{5}\left[\alpha_{5}^{2}\left(-\bar{a}+2\bar{l}t+t\right)+\lambda \zeta \left(\bar{a}-t\right)-2\right.\right.\right.\\ & & \left.\left.+\sinh\alpha_{5}\left[-\bar{a}\alpha_{5}^{4}\left(2\bar{l}+1\right)t+\alpha_{5}^{2}\left(\bar{a}\lambda \zeta t+\bar{a}-t+1\right)+\lambda \zeta \left(\bar{a}+t-1\right)\right]\right]\right\} \end{array}$$

where $G_{3}$ is an arbitrary constant.

## Appendix B

In this appendix, we report the mathematical steps taken to solve $B^{*}\left(\lambda \right)q=0.$ The scalar form of this equation reads as follows:

(A15) $$\begin{array}{ccc} {\overset{\cdot }{q}}_{M}+\zeta q_{\vartheta }+\zeta \lambda q_{T}-\frac{\left(\zeta -1\right)q_{Q}}{{\bar{l}}^{2}} & = & 0,\\ {\overset{\cdot }{q}}_{T}+q_{M} & = & 0,\\ {\overset{\cdot }{q}}_{\vartheta } & = & 0,\\ {\overset{\cdot }{q}}_{\phi }-\lambda q_{T}-q_{\vartheta }+\frac{q_{Q}}{{\bar{l}}^{2}} & = & 0,\\ {\overset{\cdot }{q}}_{Q}+q_{\phi } & = & 0, \end{array}$$

subject to the following:

(A16) $$\begin{array}{ccc} \bar{a}q_{M}\left(1\right)+q_{T}\left(1\right) & = & 0,\\ q_{M}\left(0\right) & = & 0,\\ q_{\vartheta }\left(1\right) & = & 0\\ q_{Q}\left(0\right)+\bar{l}q_{\phi }\left(0\right) & = & 0,\\ q_{Q}\left(1\right)-\bar{l}q_{\phi }\left(1\right) & = & 0. \end{array}$$

If $\bar{M},\bar{T},\vartheta ,\bar{\phi },\bar{Q}$ are solutions of (16) and (17), then the solution to (A15) and (A16) is of the form:

(A17) $$\begin{array}{ccc} q_{M} & = & \frac{1-\zeta }{\lambda {\bar{l}}^{2}}\bar{T},\\ q_{T} & = & \frac{\zeta -1}{\lambda {\bar{l}}^{2}}\bar{M}\\ q_{\vartheta } & = & 0,\\ q_{\phi } & = & -Q,\\ q_{Q} & = & \phi . \end{array}$$

We introduce the vector notation $q_{a}={\left(q_{M,}q_{T},q_{\vartheta },q_{\phi },q_{Q}\right)}^{T}$ for the solution given by (A17).
